# Africa’s unpaid outbreak frontline workers: The political economy of community health worker neglect

**DOI:** 10.4102/jphia.v17i1.1920

**Published:** 2026-09-25

**Authors:** Eric N. Ikoona, Lucy Namulemo

**Affiliations:** 1 Executive Director Office, National Public Health Agency, Freetown, Sierra Leone; 2 Foothills Community-Based Interventions, Monticello, Kentucky, United States of America

**Keywords:** community health workers, disease outbreak response, Africa, health workforce, surveillance, global health security, volunteerism, International Health Regulations, joint external evaluation

## Abstract

Community health workers (CHWs) in Africa perform indispensable functions across the outbreak response cycle. These include early case detection, contact tracing, risk communication and zoonotic interface monitoring. They depend on community embeddedness, an asset the formal health system cannot replicate in emergencies. Yet despite a substantial evidence base, more than 80% of Africa’s 1.4 million CHWs operate under volunteerism frameworks, without formal employment, salaries or enforceable protections. This is not a consequence of resource scarcity. It is a deliberate programme design choice, endorsed by governments and development partners that extracts community labour from the most precarious workers in the system without commensurate investment. We write from national public health institutions in West and East Africa, sub-regions where outbreak frequency, CHW volunteerism prevalence and weak Joint External Evaluation (JEE) surveillance scores converge. We argue that the evidence for CHW effectiveness is settled and that the policy frameworks for professionalism are in place. The principal remaining barrier is the political economy of global health security financing. The 2023 Monrovia Call to Action and 2023 Lusaka Agenda provide an actionable roadmap. The most tractable path to binding accountability is the formal incorporation of CHW surveillance capacity as a scored metric in the World Health Organization (WHO) JEE process.

## Introduction

Community health workers (CHWs) have been central to African outbreak response over the past decade. From Ebola through coronavirus disease 2019 (COVID-19) to the present mpox emergency, CHWs have repeatedly proven indispensable. Their work supports early detection, contact tracing and the community trust on which both depend. In August 2024, the Africa Centres for Disease Control and Prevention (Africa CDC) and the World Health Organization (WHO) declared mpox a Public Health Emergency of Continental Security and a Public Health Emergency of International Concern, respectively.^1^ It was the continent’s most significant formal outbreak designation since the COVID-19 pandemic. By early 2025, 23 African countries reported active transmission. The mpox outbreak’s rapid spread was largely driven by a novel Clade Ib variant that sustained community transmission in the eastern Democratic Republic of the Congo (DRC).^2^ In the same period, Rwanda contained a Marburg virus disease outbreak within 84 days of its declaration.^3^ For those of us working in national public health systems on this continent, the contrast between these two responses is not abstract. It is a demonstration, in real time, of what institutionalised community health infrastructure makes possible, and what its absence forecloses.

In Rwanda, a government-employed, nationally deployed CHW network (the *Abannyamubuzima*, one pair per village) was mobilised within 48 h of the Marburg declaration. Daily contact monitoring enabled case identification and chain mapping before transmission could extend.^3^ In parts of the eastern DRC, by contrast, community surveillance coverage has been estimated at below 30%. Laboratory turnaround times can exceed several weeks, and active conflict compounds workforce gaps. Containment has remained elusive across multiple emergencies. We acknowledge that these two responses are not a controlled comparison. Marburg and mpox differ in transmission dynamics, basic reproduction numbers and incubation profiles. Eastern DRC’s sustained conflict is itself a determinant of surveillance failure independent of CHW workforce strength. Pathogen and context matter. So does whether the workforce performing first-line detection was maintained as a standing system asset or assembled in crisis from a pool of unpaid volunteers. This opinion paper argues that neglect of the CHW workforce in Africa is structural and politically determined. The evidence and policy frameworks for change are already in place. Formalising CHW surveillance capacity as a scored metric under the International Health Regulations (IHR) offers the most tractable path to accountability.

## Functions, embeddedness and why improvisation fails

To see why the way CHWs are employed and supported matters in an outbreak, consider what these workers actually do. The evidence based on CHW contributions to outbreak response in Africa has expanded substantially over the past decade. It has been catalysed by the Ebola virus disease outbreak in the DRC from 2018 to 2020 and COVID-19. Together, this evidence reveals a cluster of seven functions. These are early case detection, contact tracing, community mobilisation, risk communication, surveillance data collection, household support and One Health zoonotic interface monitoring. All seven share a single irreplaceable quality: community embeddedness. A CHW’s value in an outbreak derives directly from relationships built before it began. It comes from knowing which households have not been seen, which gathering was attended and which burial was conducted in which manner. This knowledge cannot be acquired in a day. It cannot be outsourced to an external team arriving with a contact list.

On early case detection, studies from the West Africa Ebola response and the DRC conflict-affected zones document CHWs as the primary or sole source of early epidemiological signals.^4,5^ This was particularly evident in settings where formal infrastructure was absent or inaccessible. On contact tracing, Miller and colleagues’ multi-country study found that CHWs engaged from the outset were substantially more effective than those deployed late.^6^ The differential was explained primarily by trust erosion. Communities that had watched their CHWs excluded from the initial response did not readily accept them as contact tracers once they were needed. Sierra Leone’s ‘Operation Find Them All’ in the 2025 mpox response, and Rwanda’s Abannyamubuzima mobilisation for Marburg, illustrate the same principle. Readiness, not improvisation, determines CHW effectiveness. This is also a task better suited to CHWs than to external responders. Only locally embedded workers know which households visit specific mines, which families keep livestock, and when seasonal movements occur. This community knowledge cannot be improvised. On the emerging One Health dimension, the Rwanda Marburg report is explicit. Containment required coordinated monitoring at human-animal interfaces, including mine sites and agricultural zones, where spillover events originated.^3^

Risk communication is a further function whose importance has grown with the spread of health misinformation through social media. No mass media campaign can replicate what a trusted community resident does in a single household conversation. Community health workers’ credibility is relational and local, built over years of routine contact. Precisely this quality makes them effective counter-messengers when formal health authorities have lost, or never held, community trust.^7,8,9^

## Volunteerism as a structural choice

If CHW functions are this consequential, why do most African CHWs perform them without formal employment? The answer is structural, not technical. The determinants of CHW effectiveness are well established. They include community embeddedness, pre-positioned training, consistent supervision and formal integration into reporting structures. They also include adequate personal protective equipment (PPE), equitable remuneration and psychosocial support.^2,8,9,10^ None of these requirements is scientifically contested. As national public health practitioners, we would add a blunter observation. Most of us working in these systems know exactly what our CHW programmes need. The constraint is not knowledge; it is the structural and political architecture. For decades, that architecture has treated community health labour as a resource to be extracted without compensation rather than a workforce to be invested in.

We take seriously the argument advanced in some donor and ministry documents that volunteer status preserves community ownership of CHWs. The argument holds that paid status alters the relational legitimacy on which CHW effectiveness depends. The empirical record does not support this position. Cross-country evidence on CHW programmes suggests that effectiveness is more closely tied to consistent supervision, fair remuneration and community-led selection than to volunteer status itself.^8^ The community-ownership argument has, in many cases, served as a financing rationale more than an evidentiary one.

More than 80% of Africa’s estimated 1.4 million CHWs operate under volunteerism frameworks, without formal employment, regular salaries, health insurance or enforceable occupational protections.^10^ This is not an accident of financing scarcity. It is a prevailing programme model that has systematically chosen to deliver community health services through unpaid labour rather than through investment in a public sector workforce. Community health worker workforces in most documented African national programmes are predominantly female. The burdens of volunteerism, therefore, fall disproportionately on women, whose domestic caregiving responsibilities intensify during outbreaks. A qualitative study of CHWs during Sierra Leone’s COVID-19 response documented heavy workloads, low remuneration, lack of mental health support, and experiences of stress and stigma.^11^ These are predictable, recurring consequences of programme models that extract outbreak surveillance labour from the most precarious workers in the health system and offer nothing in return.

## The investment case

The structural choice is also, on its own terms, fiscally indefensible. Ballard and colleagues estimated the annual cost of transitioning Africa’s CHW workforce to formal, living-wage employment at approximately $3 billion – $4 billion.^10^ The total economic losses attributed to the West Africa Ebola epidemic across Guinea, Sierra Leone and Liberia alone exceeded $53 billion. That was one outbreak in three countries in 3 years. A professionalised CHW workforce, sustained at the Ballard estimate, would cost less over a decade than the economic damage of a single major epidemic. A 2025 scoping review found that most evaluated CHW programmes for HIV, tuberculosis and malaria were cost-effective compared with alternative service-delivery models, although methods and reported costs varied substantially.^12^ The arithmetic is not complicated; the political economy is.

The gap between evidence and action reflects a structural misalignment. The actors who bear the cost of CHW investment are national governments and bilateral development partners, working within constrained routine health budgets. They are not the same actors who bear the cost when community surveillance fails. That cost is borne by populations, overwhelmed health systems and the global community when containment fails and emergencies cross borders. Correcting this misalignment requires that global health security financing address CHW workforce professionalisation differently. The Pandemic Fund, IHR compliance support and African Union health security mechanisms should treat it not as a development expenditure but as an investment in a global public good with measurable returns.

## Scope and evidence base

The strongest evidence for CHW effectiveness in outbreak response derives from West and Central African settings. These include Ebola, COVID-19, the DRC outbreaks, mpox and Marburg, with a smaller body of work from East and Southern Africa. Generalisation across the continent’s varied programme designs, conflict profiles and health system maturity is not warranted by this evidence base alone. The comparisons we draw are illustrative rather than causal-comparative. We are undertaking a PROSPERO-registered systematic review to consolidate comparative effectiveness, cost and One Health evidence. Its findings will inform implementation specifics. The policy argument advanced here, however, rests on the consistency of the available evidence, not on its completeness.

## A policy agenda already in hand

Translating this case into action does not require new frameworks. It requires implementing those that already exist. We write from within national public health systems that operate under the same structural constraints described in this paper. The 2023 Monrovia Call to Action and the 2023 Lusaka Agenda represent the most operationally specific international frameworks for CHW professionalisation yet produced.^13,14^ They call for formalised employment and harmonised financing through a ‘One Plan, One Budget, One Monitoring and Evaluation’ framework. They also call for the explicit recognition of CHW outbreak-response capacity as a global health security investment. Africa CDC, the WHO Regional Office for Africa and the Pandemic Fund have each aligned with these goals in principle. Converting principle into implementation requires six evidence-supported changes.

Firstly, CHWs must be formally specified in national pandemic preparedness and response plans. Their roles, activation thresholds, reporting lines and command structures should be defined before emergencies begin. Where this has not occurred, CHWs have been excluded from initial response structures, as observed during the West Africa Ebola response. This exclusion contributed to the erosion of trust that Miller and colleagues later documented.^6^ Secondly, remuneration structures must move from volunteerism to formal employment. Outbreak-period allowances, health insurance and guaranteed PPE should be treated as minimum protections, not optional incentives. Evidence from Sierra Leone’s COVID-19 response points to the human cost of relying on CHWs without adequate institutional protection; participants reported stress and stigma while mental health support was absent.^11^ Thirdly, training must shift from emergency induction to sustained annual preparedness, including simulation-based outbreak exercises. Fourthly, CHW surveillance data must be integrated into national Health Management Information System architectures. Fewer than 40% of African countries currently achieve this integration. As a result, the continent’s richest source of community-level epidemiological intelligence remains structurally disconnected from outbreak response systems. This gap is visible in eastern DRC, where community surveillance coverage has been estimated at less than 30%.^15^ Fifthly, digital health investments must include equity safeguards for literacy, connectivity and gender-differentiated barriers to adoption. Sixthly, gender-responsive programme design and institutionalised psychosocial support must be codified as programme standards.

The accountability mechanism that gives these changes verifiable traction already exists but remains underused: the WHO Joint External Evaluation (JEE) process. Integrating CHW surveillance capacity as a scored JEE metric would create measurable country-level accountability for the first time. It would give donors, technical agencies and governments a comparable benchmark for tracking whether community-level surveillance capacity exists before the next emergency begins.

## Conclusion

What this paper adds: the case that the CHW workforce neglect in Africa is structural, not scientific, and has been argued before. What is new here is the diagnosis of the principal remaining barrier. That barrier is the political economy of global health security financing. The operational proposal that follows is to score CHW surveillance capacity as part of the WHO JEE process. Community embeddedness, defined as the capacity to detect early signals, trace exposure networks and communicate through trusted relationships, is an epidemiological asset. It cannot be manufactured under emergency conditions. It must be built in advance and maintained between emergencies. The 2024 mpox and Marburg responses have illustrated this with unusual clarity.

What should happen next: three institutional pathways are immediately available. Firstly, the Africa CDC, through its Continental Framework for Public Health Emergency Preparedness and Response, can require CHW workforce metrics as a precondition for member-state accreditation. Secondly, the African Union’s Common African Position on the Pandemic Accord can specify CHW workforce financing as a non-negotiable element of equitable pandemic preparedness. Thirdly, the WHO JEE External Review Team can pilot a CHW surveillance capacity sub-score in the 2026 evaluation cycle.

Our forthcoming PROSPERO-registered systematic review will refine implementation specifics. It will address comparative effectiveness across outbreak settings, cost-per-case-detected estimates beyond the existing scoping evidence and One Health integration. The core policy argument does not await its completion. The enabling conditions for CHW effectiveness are documented within the West and Central African evidence base, and the policy frameworks are in place. What is absent is binding accountability. Formal incorporation of CHW surveillance capacity as a scored metric in the WHO JEE process would, for the first time, provide a measurable and internationally comparable benchmark for the community health infrastructure that stands at the front line of Africa’s outbreak detection. That single institutional step would do more to translate evidence into action than any further review.
